# Massachusetts Veterinary Association

**Published:** 1891-05

**Authors:** Austin Peters

**Affiliations:** Secretary


### SOCIETY PROCEEDINGS.

*Massachusetts Veterinary Association.*—The regular meeting of the Massachusetts Veterinary Association was held at 19 Boylston Place, Boston, Wednesday evening, March 25, 1891.

President Thomas Blackwood, in the chair. Members present; Drs. Blackwood, Bunker, Emerson, Hadcock, Howard, Marshall, Peterson, C. Winslow and the Secretary. Honorary member, Dr. Stickney. Minutes of the previous meeting were read and accepted.

Moved by Dr. Peterson and seconded by Dr. Emerson, that the Secretary act as a committee of one to attend to the details of the annual meeting and dinner. Carried. Moved by Dr. Bunker and seconded by Dr. Hadcock, that the annual meeting and dinner be held at Young's Hotel. Carried.

Dr. Bunker then read a paper to which he had not given a title. It was a plea for more brave, honest, earnest co-operation among the members of the association in doing their part in the furtherance of the public good, in spite of mercenary interests or reasons of policy, as follows:

A number of years ago when the study of veterinary science was in its very infancy in this country, in fact, I might almost say before it was born, a number of practitioners of this State, of New York, and of Pennsylvania, realizing that in co-operation and union there is strength, banded together and formed a society which is in existence to-day, known as the United States Veterinary Medical Association. To this society the members were very loyal and its meetings were regularly attended, being generally held alternately in Boston and New York.

Its members comprised graduates and non-graduates. Among the latter were many men whom any school might well be proud to claim as an alumnus—workers and students they were without a doubt, as can be testified to by many of our members.

There seems to have been one purpose which from the outset pervaded these men and for the accomplishment and advancement of which, they seem ever to have worked with an enthusiasm and interest which knew no such word as fail—they kept ever at work, their motto the advancement of the profession. To these men, many of whom have laid down their scalpel for the last time and gone to their reward, the young members of the profession owe a debt of gratitude and appreciation, for it is to such men as these that we find that opening and chance for the veterinary profession which is only of recent date. To this parent society the young men, as they took up their chosen profession, attached themselves, until at last, it became a society with members all over the Union.

Its influence has been good, its standing high, but it has not received the recognition which it should have received from the public.

As the years have passed since its inception schools and colleges have been established, and have borne their fruit until the graduate practitioner is abroad in the land.

Recognizing the fact that it is well for brothers to live together in peace and harmony, the graduate members of the profession in Massachusetts were invited to meet and form a State Society. To this invitation there was a generous response, and as a result of that gathering the Massachusetts Veterinary Association was formed. Its object, the advancement of the profession and the best interest of its members.

Its mission is a noble one and one to which every member should be loyal in heart, faithful in service, and honest in service. There is for the veterinarian of to-day, a field full of opportunities, full of chances for distinction, and last but not least, a remuneration for his time—perhaps not always a princely sum, but yet, one which will at least keep the wolf from the door. There may not always be stirring times for the investigator or specialist, but as "the still sow drinks the swill" so will the quiet but earnest worker earn the laurels when the time for action comes.

A little more than thirty years ago a Massachusetts practitioner prior to sailing from England for home, purchased one or more text books to read and study during the voyage. One of these books had some wood engravings of the pathological appearances of various diseases, which were studied with much interest. Upon his arrival home, his practice was again taken up and the books for the time, laid one side. He had not been many months at home before he was called to see a new form of disease which had broken out among our herds. On visiting the herd, a diagnosis, different from his most intimate friend's was made, and which was disbelieved both by the veterinary profession and the Human school, to say nothing of comments and criticism by the press and laity, in 1859 before the Legislature. In spite of all these sources of opposition and lack of support in his opinion, Dr. Thayer kept steadily at work until he had convinced the people of Massachusetts that they had indeed in their herds that scourage of the bovine race, Contagious Pleura Pneumonia. Firm in his opinion, persistent and untiring in his work, he finally received from his brother practitioners support and co-operation. Then the Human school and laity came over and with them the legislative machine, and then the work of stamping out went on until there was not a case within the borders of the State. And all this had been done at a cost of about $65,000.

I have mentioned this case thus fully for no purpose of eulogium upon Dr. Thayer, for he needs none, his work stands ever in view of such, but simply as an example to present to this society, to show them that whenever there comes a time for similar earnest work there will be given to them that support and co-operation which is requisite for the completion of the work.

This society has got among its members many who are capable of just such work, and it would be the mission, and the object of each and every member to aid in the prosecution of such efforts.

This society was not founded with the idea of simply having a sort of monthly retreat when we can get together, swap cases, chat a while, and swap stories. Its only effort should be for the best interests of the profession, strike whom it may, it may strike you to-day, it may strike me to-morrow; but as Time is the great Leveller, so, in time, will the seeming hardships be evened up. Truth, it is said, should not always be spoken. "Honesty is the best policy." They do not always seem to be from a financial standpoint, for the time-being the best move but step right ahead, and the satisfaction of knowing that your course was right and that at no future time can your course be laid bare and proclaimed dishonest, should be far more satisfactory than the few paltry debtors. Harmony is a great thing, but there is a time when it is to be put aside and the opposite condition come in its stead.

This society should lead all efforts to advance the course of veterinary medicine or the interests of the professions in this State; its voice should be heard with no uncertain sound when questions of health bearing upon our province are brought up, or are forced into prominence.

*Now*, should always be the accepted time, no matter if it does strike in your neighborhood, perhaps disarrange some interest, for whom you render service, temporarily, how much better to be able to say, "Yes, it did exist, but we got right to work and wiped it out," than to deny its existence or to claim that those who dare to come out are wild and crazy.

This society must do its work, must do it more earnestly, and to do it thus our members must be aroused, and now as our annual meeting is upon us let us all go to work and have a grand rally from one end of the State to the other.

After Dr. Bunker ceased reading, a pause followed which was broken by the essayist, who went on to speak of the importance of our profession, and that all of its members should work for its recognition by Boards of Health, and we should unite in expressing our disapprobation of the Cattle Commissioners' innefficiency and its having no rules for the disinfection of infected premises, dealing with suspicious cases, and the like. It is time this association was up and doing in remedying existing evils, and getting better recognition of the value of our association. Dr. Winslow said that he thought that in time, owners of animals and Boards of Health should consult veterinarians upon the best course to pursue in outbreaks of contagious animal diseases, without seeking advice from the Cattle Commissioners. Dr. Stickney thought that the Cattle Commissioners' lease of life would soon be self-terminating if it continued in its present course. Dr. Marshall thought that the members of the Commission were very reasonable in their treatment of owners of stock.

Dr. Stickney said, politeness had too much to do with their action, and it makes a great deal of difference who owns the property upon which the Commission is administering. Even if they appreciate the condition of things, which they often do not, they do not have the pluck to stand up and grapple with matters as they should. One member in particular might as well be a mop, for anyone could wring him out. Dr. Howard asked the essayist if he recommended our acting as individuals, or as an association? Dr. Bunker advised acting as individuals, but as members of the Massachusetts Veterinary Association. Dr. Howard said, that our association had investigated the Cattle Commission once, but he did not see that much had come of it. Dr. Marshall moved that the essayist be given a vote of thanks. Seconded and carried.

Dr. Hadcock reported a case of Melanosis in a roan horse. First noticed a swelling of the abdomen on the near side, one Thursday; gave a carthartic ball which acted nicely the next day (Friday), but he did not see him until Saturday. Saturday, the abdomen was very much swollen, and horse had colicy pains. Sunday morning, Dr. Blackwood saw him in consultation, abdomen was very much distended with fluid; it was proposed to tap him, but after talking it over decided not to, horse showing symptoms of Enteritis. He died Sunday night, post-mortem made Monday morning; the abdomen contained a quantity of dark-colored fluid and melanotic tumors were found in the mesentary, liver and spleen. The mesentary looked like a huge black sponge.

Dr. Peterson reported a case of fatal Epistaxis in a heifer, at Sudbury. When Dr. Peterson arrived at the farm where the heifer was owned, he found a small stream of what looked like arterial blood flowing from both nostrils; she had bled about a bucketful. He injected six ounces of tincture of perchloride of iron into her nostrils, used cold water and tried plugging, but could not check the hemorrhage, she had lost two bucketfuls of blood by the time he left, and died the next day. He did not have an opportunity of making an autopsy, but she was opened by her owner, who said that he found no lesions to account for the trouble, still a professional man might have.

Dr. Bunker spoke of a horse which came under his observation where pieces of sponge had been plugged up both nostrils to stop the noise of whistling, which it effectually did.

Meeting then adjourned.

AUSTIN PETERS, *Secretary.*

